# Implementation of a peer health navigator program for patients at risk of frequent hospitalisation

**DOI:** 10.1186/s12877-025-06945-y

**Published:** 2026-02-05

**Authors:** Rebecca L Jessup, K Stockman, D Nguyen, C Haywood, M Suleiman, S Thomas, Johanna Hayes, Anne-Marie Fabri, D Campbell

**Affiliations:** 1 School of Allied Health, Human Services and Sport, La Trobe University, Bundoora, Melbourne, Australia; 2 Staying Well and Hospital Without Walls Program, Northern Health, Epping, Melbourne, Australia; 3 School of Rural Health, Monash University, Warragul, Australia; 4 Faculty of Art, Design and Architecture, Monash University, Clayton, Melbourne, Victoria Australia; 5 Austin Health Department of Medicine, Austin Health, Heidelberg, Australia; 6 Department of Medicine, University of Melbourne, Parkville, Australia; 7 Victorian Virtual Emergency Department, Northern Health, Epping, Melbourne, Victoria Australia; 8 Office of Research, Northern Health, Epping, Melbourne, Australia

**Keywords:** Peer health navigation, Frequent hospitalisation, Transitional care, Frailty, Health-related quality of life, Older adults

## Abstract

**Background:**

Individuals at risk of frequent hospitalisation often experience complex health and social challenges. Peer health navigation programmes have emerged as a promising strategy to support these patients, improve continuity of care, and reduce hospital use. The strategy has strong alignment with the transition of care management model.

**Objective:**

To describe the baseline demographic characteristics, self-reported health status, and frailty of patients enrolled in the Northern Patient Watch (NPW) program. NPW employs peer health navigators, under the supervision of health professionals, to support individuals at high risk of recurrent hospital admissions.

**Methods:**

This observational study examined all patients who enrolled in the NPW programme between November 2021 and October 2024. Baseline data were collected on demographics, health-related quality of life (EQ-5D-5 L), and frailty (Rockwood Clinical Frailty Scale). Descriptive statistics were used to summarise the findings.

**Results:**

A total of 650 patients were enrolled. The mean age was 70 years, and 56% were female. One-third lived alone, and 29% spoke a language other than English at home. The mean EQ-5D-5 L index score was 0.72, with a mean EQ-VAS of 58.9, indicating moderate health-related quality of life. Moderate issues were frequently reported across all EQ-5D-5 L dimensions, particularly in mobility, pain, and mental health. Two-thirds of participants were classified as vulnerable or frail.

**Conclusion:**

Patients who enrol in peer health navigation programmes such as NPW are typically older adults with moderate but not severe health challenges. These individuals may be particularly well positioned to benefit from early, tailored support that can prevent further deterioration. Findings highlight the potential value of peer navigation programmes in engaging at-risk populations before health crises occur.

## Introduction

Public hospital spending is the fastest-growing area of government expenditure [1]. A small cohort of complex patients account for a disproportionate share of healthcare spending [2]. Faced with a fragmented and hard-to-navigate health system, these individuals are often caught in a cycle of care that fails to meet their needs, leading to repeated hospital admissions, prolonged stays and potentially preventable health crises [3]. Health and social factors including multimorbidity, mental health challenges, social isolation and loneliness, and limited access to preventive care further increase the risk of recurrent hospitalisation [4–6]. Specifically, preventable hospitalisations are a major contributor to rising health costs, placing strain on acute care capacity, and highlighting the need for community-based approaches that address both health and social determinants of wellbeing. Addressing the causes of high health care utilisation through the implementation of proactive, tailored strategies that address these determinants has the potential not only to improve health outcomes for these individuals but may also enhance the efficiency and sustainability of the broader health system long term [7, 8].

For this reason, many health systems worldwide are implementing comprehensive, person-centred programs that aim to reduce avoidable hospital use. These integrated care initiatives aim to strengthen wellbeing support, overcome access barriers, and improve continuity of care [9]. They often support patients at one of the most vulnerable points in their care journey – the transition of care from hospital to home. Integrated care initiatives at discharge may focus on supporting patients to follow treatment plans and medication regimens, attend follow-up appointments, and to access community-based services that meet their preventative and proactive care needs [10, 11]. In addition, they aim to improve communication between care providers. In Australia, these approaches are increasingly prioritised through policy initiatives that emphasise care closer to home and the importance of equitable access to preventive and transitional care.

One well-established framework underpinning these efforts is the Transitions of Care Management (TCM) framework [12], developed in the United States to ensure safe and coordinated movement of patients from hospital to community care. The TCM framework emphasises timely follow-up, improved communication between providers, and active patient engagement to reduce preventable readmissions [12]. Although the framework is primarily formalised within the United States Medicare system, its principles have been widely adopted across international integrated-care initiatives. The Australian Commission on Safety and Quality in Health Care, recognising that poor transitions of care are associated with increased adverse events, readmissions, and medication errors, has prioritised improving transition processes as a national safety and quality priority.

Improved monitoring of patients after discharge, patient education, and care coordination have emerged internationally as strategy that can successfully improve the transition of care and reduce hospital readmissions [13–16]. Providing patients with targeted support from peer health navigators is one method that has been used to address the higher level of care that may be required for some patients as they transition from hospital to home [17]. Peer and lay health navigators are individuals without a health care occupational background who often (though not always) have lived experience of managing a health condition, navigating health and social services, or overcoming structural barriers similar to those faced by the patients they support [18–20]. This shared experience allows them to build rapport and trust in ways that traditional clinical roles may not. Peer and lay navigators work alongside patients to provide practical assistance with navigating the health system, including support for attendance to appointments, clarifying treatment plans, providing wellbeing support, and helping patients overcome access barriers to care including transport and accommodation [21]. They also play an important role in bridging gaps in health literacy and cultural understanding, particularly for patients from diverse backgrounds or those who may feel marginalised within traditional healthcare settings [22]. By offering emotional support, education, and continuity, peer navigators build trust and empower patients to take a more active role in managing their health, which in turn can reduce unnecessary emergency visits and readmissions.

The aim of this study was to describe the characteristics of patients with complex and chronic conditions who have been identified as at risk of readmission, and who have been enrolled in a peer navigator program.

## Methods

### Design

A descriptive observational design was used to provide an overview of the characteristics of patients who voluntarily enrolled in the peer health navigator programme.

### Setting and context

Northern Health (NH) is the main provider of acute healthcare services in Melbourne’s outer northern metropolitan region. This community is culturally and linguistically diverse, with more than 40% of the population born overseas and more than 185 languages spoken. The area faces socioeconomic challenges, including lower income levels, reduced educational attainment, and lower health literacy, as well as higher unemployment rates compared to Victorian averages [23].

### Intervention

**Fig. 1:**
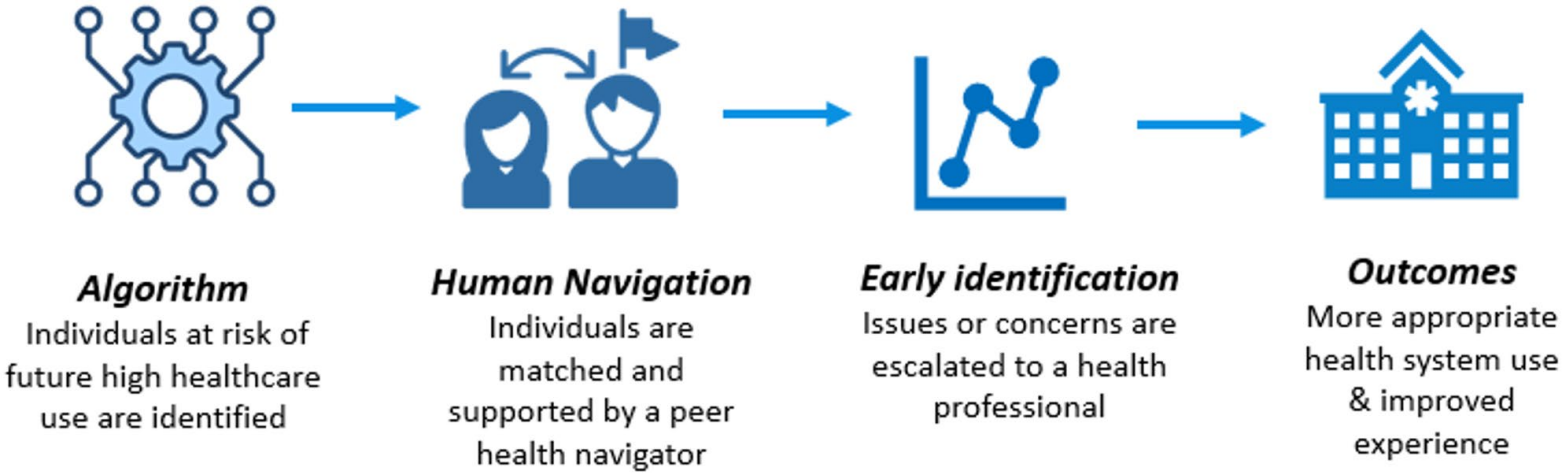
Overview of northern patient watch model of care

Northern Patient Watch (NPW) is a care model involving regular health monitoring by peer health navigators supported by health professionals (Fig. 1). The model involves identifying patients at risk of frequent potentially avoidable hospital admissions via an algorithm [24]. The algorithm identifies patients at high risk of frequent hospitalisation by using an unplanned index admission as a trigger and combining diagnostic and demographic information to generate a risk score for three or more admissions within 12 months. Variables include age, recent hospital and emergency presentations, chronic conditions, smoking status, and place of residence. Eligible patients are then approached and offered enrolment in the program. The navigators monitor participants’ health over a minimum period of three months, with the potential for ongoing support extending to several years. The navigators speak a mix of languages, including Arabic, Italian, Turkish and Macedonian and they conduct monitoring phone calls in the patient’s preferred language or with the use of an interpreter which has been found to improve engagement with care [25]. Monitoring is conducted weekly by the navigator using structured self-rated health checks via telephone supported by the Smart Health and Patient Journey Record (PaJR) [26] systems for contact management and decision support. Patients or their carers can call the service during business hours for assistance.

When there are signs of health deterioration triggered by PaJR, or other concerns, the patient is escalated to the care of a supervising health professional (usually nursing or allied health) who provides care management services. This may include coordinating patient-centred activities to support self-management and addressing barriers, such as transport or respite needs, to ensure timely access to appropriate care. These health coaches encourage patients to understand their health conditions, self-manage where possible, and seek additional support or intervention when needed. Health coaches collaborate closely with Northern Health clinicians and programmes, as well as external service providers, to deliver comprehensive support. The patient’s general practitioner plays a central role in coordinating clinical care within the community, and is kept informed and involved in all care provided by NPW.

A recent evaluation of the NPW program used a pragmatic propensity-score matching design to compare outcomes for patients who enrolled in the program with those who were eligible but declined participation [27]. Matching was based on demographic and clinical variables, including age, sex, comorbidities, and prior hospital utilisation, to minimise selection bias. The analysis demonstrated that patients supported by peer health navigators experienced approximately half the number of hospital presentations and bed-days compared with matched non-participants, as well as increased attendance at outpatient appointments. These findings provide early evidence that the NPW model of peer health navigation can reduce hospital resource utilisation and strengthen continuity of care for adults at high risk of readmission.

### Population

This study reports on all eligible NPW patients who were enrolled in the program from 1st November 2020 (inception) until the 30th October 2024. Enrolment in the program was voluntary, so only individuals who consented to participate were enrolled.

Patient eligibility for the program is via identification using the risk algorithm. The algorithm was developed using administrative datasets as part of the Health Links Chronic Care (HLCC) program, a Victorian Department of Health and Human Services capitated funding initiative that targeted those patients with chronic and complex care needs who were at high risk of three or more unplanned admissions in the subsequent 12 months [28]. The intent was to incentivise Health Services to better support these vulnerable patients, selected based on the risk-based selection algorithm, in the community and, as a consequence, reduce the number of potentially avoidable hospitalisations. The NPW program was set up as part of this program.

The HLCC-derived algorithm uses an unplanned index admission as a triggering event and then combines diagnostic information from that admission with demographic information to create a ‘risk score’ for the probability of three or more admissions in the next 12 months. Admission and demographic information include age, number of unplanned admissions in the past six months, emergency department visits in the past three months, hospitalisations due to certain progressive conditions and co-morbidities (such as asthma, kidney disease, chronic osbructive pulmonary disease, heart disease, rheumatoid arthritis), smoking status, and type of residence (aged care or private residence). Patients that have an index admission and score above a threshold value determined by logistic analysis of historical data were eligible to be included in the NPW program [28]. A model with higher sensitivity and specificity was subsequently developed by our research team using administrative and clinical data (titled the Hospital Unplanned Readmissions Tool or HURT) [29], and a combination of both algorithms were used to identify eligible patients for NPW based on risk for subsequent admission on three or more occasions in the next 12 months.

### Data collection and measures

Demographic and health-related data were collected for all participants, including age, sex, and body mass index (BMI). Social and living circumstances were recorded, comprising living arrangement, language spoken at home, and sources of social support. Cultural and linguistic diversity was captured through identification of languages other than English spoken at home, with participants able to nominate multiple languages. Socioeconomic and functional characteristics included main source of income, mode of transport, and mobility status (independent, requiring a gait aid, or dependent). Health-related variables included self-reported vision and/or hearing impairment, tobacco smoking, and alcohol consumption. These data were obtained through participant self-report at enrolment and cross-checked, where possible, against existing clinical or administrative records. In addition, all enrollees completed the EQ-5D-5L to determine health status, and the Rockwood Frailty Scale to provide a frailty score. Each of these measures are validated and are described in detail below.

#### EQ-5D-5 L

Health status was assessed using the EQ-5D-5L, a widely-used, multi attribute instrument developed by the EuroQol Group. It includes two components: a descriptive system and a visual analogue scale (VAS). The descriptive system captures self-reported health across five dimension: *(1) mobility*,* (2) self-care*,* (3) usual activities*,* (4) pain/discomfort*,* and (5) anxiety/depression* [30]. Each dimension has five levels of severity ranging from no problems to extreme problems or inability. Responses are converted into a single utility index score using a country-specific value set; in this study, the Australian algorithm was applied. Index scores range from values −0.59 (health states considered worse than death) to 1 (full health), with higher scores indicating better health-related quality of life. Participants also rated their overall health on the day of enrolment assessment using the EQ VAS, a vertical scale from 0 (worst imaginable health) to 100 (best imaginable health). The EQ-5D-5 L provides both a preference-based utility score and an individual’s subjective assessment of health, enabling a comprehensive view of health status.

#### Rockwood clinical frailty scale

The Rockwood Clinical Frailty Scale is a clinical measure of an individual’s fitness and frailty based on physical and cognitive abilities, categorising them into nine levels: *(1) Very Fit*,* (2) Well*,* (3) Managing Well*,* (4) Vulnerable*,* (5) Mildly Frail*,* (6) Moderately Frail*,* (7) Severely Frail*,* (8) Very Severely Frail*,* (9) Terminally Ill* [31]. This index is particularly useful for assessing older adults, predicting healthcare needs, and guiding treatment planning. It integrates subjective assessments with clinical measures to create a comprehensive picture of vulnerability.

### Statistical methods

Data were prepared in csv files by the Northern Health data warehouse with statistical analyses conducted using open R source statistical software. We used descriptive statistics to provide proportions and frequencies for demographic, clinican and social variables. Means, medians, and ranges were used to describe continuous variables including age and BMI. EQ-5D-5 L scores were calculated using the Australian value set, and summarised across dimensions including mobility, self-care, and pain, while also calculating mean and median EQ-VAS scores. Frailty scores were summarised using frequency distributions, highlighting the proportion of patients in each frailty category.

## Results

A total of 650 patients were enrolled in the patient watch program during the study period. Table 1 provides an overview of the demographics of enrolees. The majority lived with others, with just over ¼ living alone. More than half were independent in mobility (*n* = 353, 54.3%), though 273 (42.0%) required a gait aid, and 8 (1.2%) were dependent. 258 (39.7%) reported vision issues, and 143 (22.0%) reported hearing difficulties. Sources of support primarily came from family (398, 61.2%) and spouses/partners (*n* = 258, 39.7%). Only 42 (6.5%) reported that they had no support. Regular alcohol consumption was reported by almost ¼ of participants (*n* = 157, 24.5%), tobacco use by 108 (16.8%), and drug use (excluding alcohol or tobacco) by 9 respondents (1.4%).

**Table 1:** Characteristics of enrolees of the Northern Patient Watch program

Characteristic (*n* = 650)	*n*
Age mean (IQR range)	70 (62–80)
Female	361 (56%)
BMI mean (IQR)	30 (25–33)
Living Arrangement	
Lives alone	172 (27%)
Lives with others	478 (73%)
Language other than English spoken at home	190 (29%)
Top 5 languages other than English	
Arabic	48 (7%)
Italian	43 (7%)
Greek	18 (2%)
Turkish	15 (2%)
Macedonian	14 (1%)
Sources of support (more than one may apply)	
Spouse/ partner	258 (40%)
Family	398 (61%)
Friend	60 (9.2%)
None	42 (6.5%)
Mode of transport (more than one may apply)	
Self-drive	345 (53%)
Driven by others	374 (58%)
Public transport	57 (9%)
Mobility	
Dependent	8 (1%)
Gait aid required	273 (42%)
Independent	353 (54%)
Unknown	16 (2.5%)
Main source of income	
Pension (Age, Disability)	498 (77%)
Full-time Work Partner (Spouse)	29 (5%)
Part-time Work	26 (4%)
Superannuation/Investments	28 (4%)
Newstart (Jobseeker, etc.)	32 (5%)
Other	22 (3%)
Vision issues	258 (40%)
Hearing issues	143 (22%)
Smokes tobacco	108 (17%)
Regularly consumes alcohol	157 (24%)

The EQ-5D-5L was completed by 632 enrolees. The EQ-5D-5L index scores ranged from − 0.293 to 1.00, with a mean of 0.72 (SD 0.25), with the majority of enrolees experiencing health limitations. EQ-VAS mean score was 58.9 (SD 21.3) indicating that participants view their current health as just under 60% of the best possible health they can imagine.

**Fig. 2:**
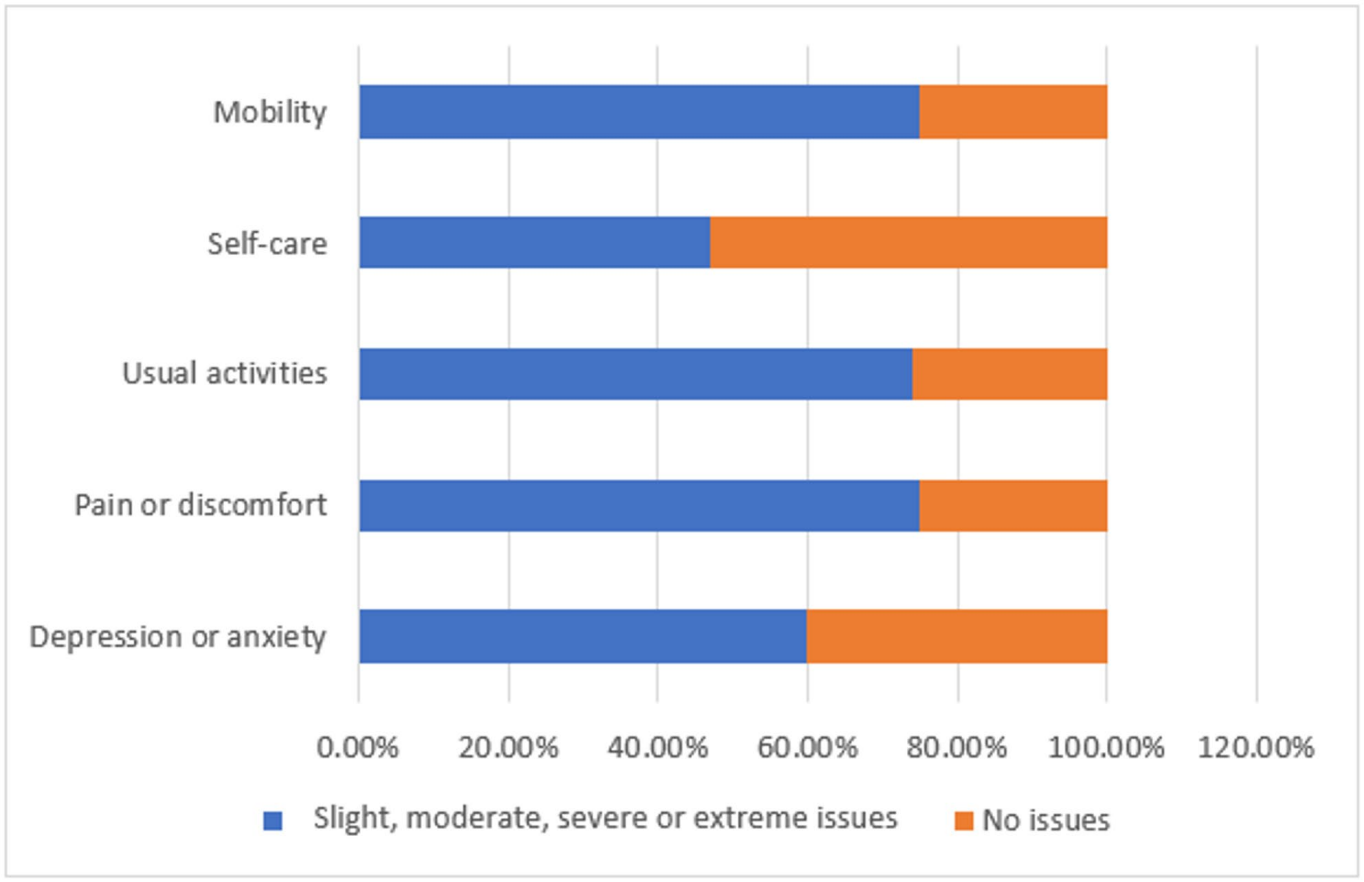
Distribution of EQ-5D-5L scores across mobility, self-care, usual activities, pain/ discomfort, and depression/ anxiety dimensions

The majority of participants reported some issues with their mobility, self-care, usual activities, pain or discomfort, and depression or anxiety on the EQ-5D-5L (Fig. 2). In the mobility domain, 215 (34%) reported moderate problems and 177 (28%) reported slight problems walking about, while only 1/4 (*n* = 158) reported no problems. For self-care, over half of respondents (*n* = 334, 53%) reported no problems washing or dressing themselves, however 120 (19%) reported moderate difficulties and 139 (22%) reported slight difficulties. In terms of usual activities, moderate (*n* = 208, 33%) and slight (*n* = 139, 22%) problems were again most common, with only 26% reporting no issues. The pain or discomfort domain followed a similar pattern, with 34% experiencing moderate pain, 158 (25%) reporting slight pain, and another 158 (25%) reporting no pain. For depression or anxiety, 252 (40%) of respondents indicated they were not anxious or depressed, while 170 (27%) were slightly and 164 (26%) were moderately affected. Only a small proportion of participants in each domain reported extreme or severe problems.

**Fig. 3:**
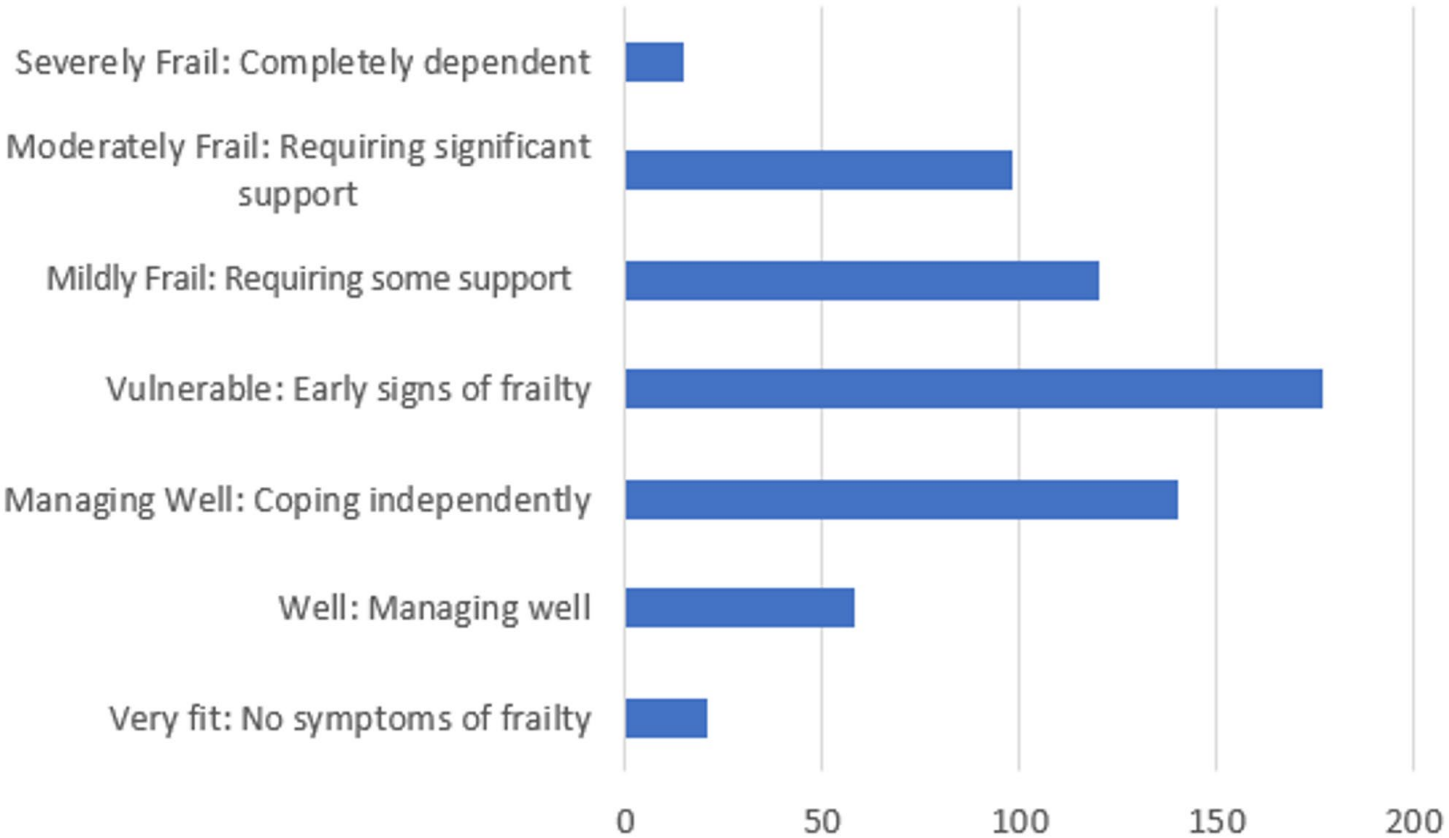
Overview of distributions of Rockwood Clinical Frailty Scale scores

The Rockwood Clinical Frailty Scale was completed for 631 patients. A median score of 4 revealed that a significant portion of the group falls into vulnerable or higher categories indicating a high level of frailty in the group (Fig. 3). When combined those classified as vulnerable (*n* = 177), mildly frail (*n* = 120), “moderately frail” (*n* = 98), severely frail (*n* = 15), and very severely frail (*n* = 2), equate to 65% of the cohort experiencing varying degrees of frailty or health challenges. This suggests that most enrolees are at an elevated risk of functional decline and other health complications. A smaller proportion of participants (*n* = 140, 22.2%) were classified as managing well, indicating that a substantial portion of the group requires additional support in managing their health and daily activities.

## Discussion

This study provides an overview of the characteristics of patients enrolled in a peer navigator program specifically designed to target individuals at risk of future hospital admissions. The findings highlight the program’s reach, with enrolees representing a diverse, predominantly vulnerable population. Two in three enrolees were classified as either vulnerable or frail (mild to severe) on the Rockwood Clinical Frailty Scale, and almost 1/3 lived alone, demonstrating the health and social challenges experienced by this cohort. In congruence with the frailty scores, scores on the EQ-5D-5L indicated that the majority of enrolees had mild to moderate impairments across one or more health dimensions, including difficulties with mobility, self-care and usual activities. The majority of enrolees also experienced slight or moderate daily pain and many reported experiencing anxiety and depression.

Peer navigator programs may represent a promising intervention for high resource users to address barriers to care including health literacy and communication issues, care fragmentation, and access to support services. A trial of peer navigators to support patients with discharge preparation, medication management, scheduling of follow-up appointments, communication with primary care, and symptom management found that older enrolees benefited most, and that they were significantly less likely to experience hospital readmissions if they were supported by a navigator [18]. Peer navigators, often individuals with shared lived experiences or cultural backgrounds, build trust and foster patient engagement by providing emotional support, guidance, and practical assistance. The World Health Organisation commissioned a policy brief in 2022 that identified there is sufficient research to suggest that health navigators are a promising solution to bridging health equity and access gaps, but that due to differences in health systems between countries, results of trials may not always be transferrable [17]. It is therefore important that we build the evidence for peer health navigators within the Australian health system.

The NPW program aligns conceptually with established transitions of care frameworks [12]. NPW extends traditional approaches that focus on clinical handover and information transfer between providers by incorporating peer health navigators who provide relational and practical support to patients. Embedding navigators into this pathway, the program strengthens continuity of care by helping patients navigate appointments, medications, and social supports, while addressing practical and psychosocial barriers that can hinder recovery after discharge. Although this study describes participant characteristics rather than program outcomes, the findings highlight the potential for peer health navigators to enhance the person-centred and relational aspects of transitional care, particularly for individuals with complex needs or limited health system literacy. The baseline characteristics of NPW enrolees also provides new insights into the specific patient population that is most likely to accept enrolment into, and therefore benefit from, a peer navigation program to reduce hospitalisation rates in high risk populations. Our finding that enrolees were generally older, with a higher prevalence of anxiety and depression, aligns with the international literature [4]. In addition, the majority of enrolees exhibited moderate rather than severe health challenges, indicating that the type of patients who accept enrolment into peer navigation programs are not severely impaired but are facing enough health difficulties to warrant additional support. This suggests that the patients likely to benefit most from peer navigator programs are those with moderate health complaints who are motivated to engage in the program.

The moderate level of frailty observed in many participants indicates that the NPW program might also play an important role in early identification and escalation of health deterioration, allowing for timely interventions to prevent hospitalisations. A propensity score matching study we have previously conducted in a subset of NPW patients who have completed the program found that patients enrolled in the program experienced half the number of hospital presentations and half of the bed-day usage, as well as increased outpatient appointment attendance, compared to those who declined enrolment [27]. When combined, these findings suggest that peer health navigation programs may be particularly effective when targeted toward individuals at a tipping point—those who are not yet severely ill but are beginning to experience challenges that place them at risk of hospitalisation and are motivated to engage. By intervening at this stage, such programs can offer timely support that empowers patients to manage their health more effectively, access appropriate services, and potentially avoid further deterioration. These insights have important implications for the design and targeting of future navigation programs, highlighting the value of early engagement and the need to tailor support to individuals with moderate yet escalating health needs.

Around 30% of patients spoke a language other than English at home, highlighting the importance of having peer navigators from a range of diverse cultural and linguistic backgrounds. Bicultural health navigators play an important role in improving access to care by bridging significant cultural, linguistic, and systemic gaps between healthcare providers and patients from culturally and linguistically diverse communities [32]. These communities often face unique barriers to healthcare, including language difficulties, cultural misunderstandings, lack of access to culturally appropriate reading materials, and a lack of familiarity with the healthcare system, which can lead to delayed diagnoses, miscommunication, and inequitable treatment. Bicultural peer navigators help mitigate these challenges by fostering trust, improving communication, and advocating for culturally appropriate care. Our strong engagement with CALD populations suggests our model improved patient engagement and may in the future contribute to reducing healthcare disparities for those enrolled.

One of the major strengths of this study is the broad diversity of the population, a group that we believe is representative of many patients dealing with the chronic and complex health issues increasingly faced by older people living in high income countries. For this reason, it is likely that the results are translatable to hospitals servicing similar lower socioeconomic areas in Australia and abroad as the challenges are similar. However, this study only includes patients who chose to enrol in the programme, and they may have been more motivated or have had higher health literacy compared to those who did not enrol. The demographic and self-reported health status and frailty data alone do not offer a comprehensive understanding of the challenges faced by participants: personal experiences, cultural factors, and specific barriers to care would provide richer insights. These limitations should be considered when interpreting the findings, and future research could address these gaps to better evaluate the programme’s impact.

## Conclusion

A peer health navigation program is a feasible model to address preventable hospital admissions. Our baseline data provides new evidence on the demographic and self-reported health status and frailty of people who have enrolled in a peer health navigation program that aims to reduce preventable hospital admissions. The work highlights the importance of targeting the right individuals for peer navigation programs: specifically those individuals who, while not at the extreme ends of health need, will benefit from personalised support. By focusing on those with moderate health challenges, peer navigation programs may help improve quality of life, enhance patient engagement, and prevent further complications. This baseline data sets the stage for future research exploring the effectiveness of peer navigation in improving outcomes for this patient population.

## Data Availability

The datasets used and/or analysed during the current study are available from the corresponding author on reasonable request.

## Abbreviations

HLCC: Health Links Chronic Care
NH: Northern Health
NPW: Northern Patient Watch
PaJR: Patient Journey Record
